# FIDMT-GhostNet: a lightweight density estimation model for wheat ear counting

**DOI:** 10.3389/fpls.2024.1435042

**Published:** 2024-10-10

**Authors:** Baohua Yang, Runchao Chen, Zhiwei Gao, Hongbo Zhi

**Affiliations:** School of Information and Artificial Intelligence, Anhui Agricultural University, Hefei, China

**Keywords:** FIDMT, GhostNet, counting, convolutional neural network, wheat

## Abstract

Wheat is one of the important food crops in the world, and the stability and growth of wheat production have a decisive impact on global food security and economic prosperity. Wheat counting is of great significance for agricultural management, yield prediction and resource allocation. Research shows that the wheat ear counting method based on deep learning has achieved remarkable results and the model accuracy is high. However, the complex background of wheat fields, dense wheat ears, small wheat ear targets, and different sizes of wheat ears make the accurate positioning and counting of wheat ears still face great challenges. To this end, an automatic positioning and counting method of wheat ears based on FIDMT-GhostNet (focal inverse distance transform maps - GhostNet) is proposed. Firstly, a lightweight wheat ear counting network using GhostNet as the feature extraction network is proposed, aiming to obtain multi-scale wheat ear features. Secondly, in view of the difficulty in counting caused by dense wheat ears, the point annotation-based network FIDMT (focal inverse distance transform maps) is introduced as a baseline network to improve counting accuracy. Furthermore, to address the problem of less feature information caused by the small ear of wheat target, a dense upsampling convolution module is introduced to improve the resolution of the image and extract more detailed information. Finally, to overcome background noise or wheat ear interference, a local maximum value detection strategy is designed to realize automatic processing of wheat ear counting. To verify the effectiveness of the FIDMT-GhostNet model, the constructed wheat image data sets including WEC, WEDD and GWHD were used for training and testing. Experimental results show that the accuracy of the wheat ear counting model reaches 0.9145, and the model parameters reach 8.42M, indicating that the model FIDMT-GhostNet proposed in this study has good performance.

## 1 Introduction

Wheat is a widely planted crop in the world, and its yield and quality are directly related to global food security and the development of agricultural economy (Atamanyuk et al., 2023). Wheat ears, as the reproductive organs of wheat, are the key to reflecting the growth status, yield potential and quality characteristics of wheat. The precise positioning and accurate counting of wheat ears is an important link in agricultural production, which not only involves real-time monitoring of wheat growth status, but also is a core indicator for predicting wheat yield. Through high-precision positioning and counting technology, agricultural scientists and researchers can more accurately grasp the wheat growth, reproductive ability, and yield potential of wheat, thereby providing scientific basis for agricultural management, breeding optimization, and yield prediction. In addition, the positioning and counting of wheat ears are also of great significance to wheat breeding work (Hasan et al., 2018). By comparing the number and distribution of wheat ears under different varieties or different treatment conditions, breeders can screen out more advantageous germplasm resources and develop wheat varieties that are more suitable for the local growth environment and market demand (Simão et al., 2023).

Traditional wheat counting methods are highly dependent on the experience and visual judgment, resulting in inaccurate and unrepeatable counting results. With the widespread application and rapid development of artificial intelligence, deep learning has made significant progress and breakthroughs in the field of wheat ear counting research. Compared with traditional machine learning-based methods, its performance has been significantly improved. Deep learning models can handle more complex data patterns and optimize their performance through training on large amounts of data (Zou et al., 2023). In terms of wheat ear counting, deep learning is mainly based on three methods, target detection, image segmentation and density map estimation.

Methods based on target detection mainly use deep learning models to identify and locate wheat ears in images. Many scholars have trained detection models to identify the characteristics of wheat ears and mark the position of each wheat ear in the image. Madec et al. (2019) used the Faster R-CNN model to detect and count wheat ears on high-resolution wheat images. In fact, the advantage of this method is that it can accurately identify and locate each wheat ear. Li and Wu (2022) proposed an improved YOLOv5 algorithm based on shallow features. By adding four times downsampling to the feature pyramid to capture the characteristics of micro-wheat ears, an attention mechanism was added to the network to realize the detection and counting of wheat ears. Yang et al. (2021) used YOLOv4, which added a channel attention mechanism and a spatial attention mechanism, to suppress irrelevant background information, increase the expression ability of wheat ear features, and detect and count wheat ears in different data sets. Wang et al. (2021b) proposed an improved EfficientDet-D0 target detection model for wheat ear counting. Research shows regardless of the single-stage or two-stage detection network, it can achieve good results in wheat ear detection and counting. However, when wheat planting density is high and leaves are severely blocked, the target detection algorithm may cause false detections or missed detections, resulting in inaccurate counting results. Therefore, it is necessary to build a more robust model.

The counting method of wheat ears based on image segmentation is to segment the wheat ears in the image from the background or other objects. This method usually uses a deep learning model to learn pixel-level features in the image to accurately segment wheat ears and background. Through the segmented image, the number of wheat ears can be calculated. For example, Zhang et al. (2022a) used the semantic segmentation model Wheat-Net to segment and count wheat ears under complex backgrounds in the field. Wang et al. (2021a) proposed a semantic segmentation regression network SSRNet to count wheat ears in remote sensing images. Ma et al. (2020) proposed a model based on semantic segmentation, EarSegNet, which effectively improved the segmentation accuracy and efficiency of winter wheat ears. Xu et al. (2023) proposed the CBAM-HRNet model for wheat grain segmentation and counting. Misra et al. (2020) proposed a segmentation model SpikeSegNet to implement counting of wheat ears. Dandrifosse et al. (2022) developed the DeepMAC segmentation model to achieve wheat ear segmentation and density counting. Although the above segmentation models can improve the accuracy of wheat ear counting to a certain extent, due to the influence of environmental factors such as lighting, occlusion, and shadow, some wheat ears may be mistakenly segmented or missed, thus affecting the accuracy of counting. However, due to the diversity of wheat planting environments and growing conditions, it is difficult to ensure the generalization ability of the segmentation algorithm in different scenarios.

The method based on density estimation uses a deep learning model to estimate the density of wheat ears in the image. It does not require precise identification and positioning of each wheat ear, but estimates the overall distribution of wheat ears in the image. In addition, density map-based methods have greater flexibility and generalization capabilities and can be flexibly applied to counting tasks of different scales and scenarios. For example, Bao et al. (2020) used the CSRNet model based on density estimation and suitable for target counting in dense scenes to count wheat ears. Sun et al. (2021) optimized the CSRNet model to count wheat ears on the global wheat data set. Wu et al. (2023) proposed the DM-Net model to estimate and count the density of wheat ears. Ma et al. (2022) proposed a transfer learning model EarDensityNet based on a fully convolutional neural network to count wheat ears. Lu et al. (2022) proposed a TasselNetV3 model based on density estimation to implement wheat counting. Since the density map can reflect the spatial distribution of objects, it can better handle the occlusion problem between objects. Research shows that density map-based methods have significant advantages in the field of wheat counting and can overcome the limitations of traditional methods and improve counting accuracy and efficiency. Although the above-mentioned counting model based on density estimation has achieved certain results, it still has shortcomings in terms of training time, parameter redundancy, processing high density, and adapting to complex scenes. Therefore, wheat counting based on density plot regression still faces great challenges.

To this end, a wheat ear density estimation model based on lightweight convolutional neural network is proposed in this study, which can better alleviate the above-mentioned problems. The purpose of this study is: (1) to solve the problem of different size of wheat ears, GhostNet is proposed as the feature extraction network, which can generate multi-scale feature maps through its unique Ghost module; (2) to improve the counting accuracy caused by dense wheat ears, FIDMT based on point annotation is introduced as the baseline network; (3) to obtain more characteristic information of small target wheat ears, a dense upsampling convolution module is proposed; and (4) to overcome the background noise or interference of wheat ears, a local maximum detection strategy is adopted to realize automated processing of wheat ear counting.

## 2 Materials and methods

### 2.1 Data collection

#### 2.1.1 Data sources

Wheat images were collected the National Modern Agriculture Demonstration Zone (31°29′26″N, 117°13′46″E) located in Guohe Town, China. The shots were taken on May 7, 2021 and May 17, 2021 using the rear main camera (48 million pixels) of Huawei’s nova5pro mobile phone. When collecting each image, the camera lens was kept at a distance of about 30cm directly above the wheat ears. A total of 500 top-view images of wheat with a resolution of 3024 × 3024 pixels were collected and record them as wheat ear counting (WEC) data set, which contains many samples with different light intensities, different densities, and different periods. Among them, there are 313 images of wheat in the filling stage and 187 in the mature stage.

To ensure the diversity and representativeness of wheat ears in the data set and enhance the universality and robustness of the model, this article added 500 images from the global wheat detection data set (GWHD, http://www.global-wheat.com) with a resolution of 1024 × 1024 pixels. This data set contains wheat data from multiple countries and different growth stages. In addition, a total of 500 clear wheat images from 10 countries in the GWHD dataset were selected, including Australia, Canada, China, France, Japan, Mexico, Sudan, Switzerland, the United Kingdom, and the United States. Wheat data from the wheat ears detection dataset (WEDD) was also added, and a Sony ILCE-6000 digital camera was used to maintain a distance of 2.9 meters from the ground for overhead photography. To reduce the number of parameters and increase the computing speed in the subsequent model training process, the wheat images with an original size of 6000 × 4000 pixels in the data set were cropped, and 200 wheat images with a resolution of 3072 × 3072 pixels were obtained.

**Figure 1:**
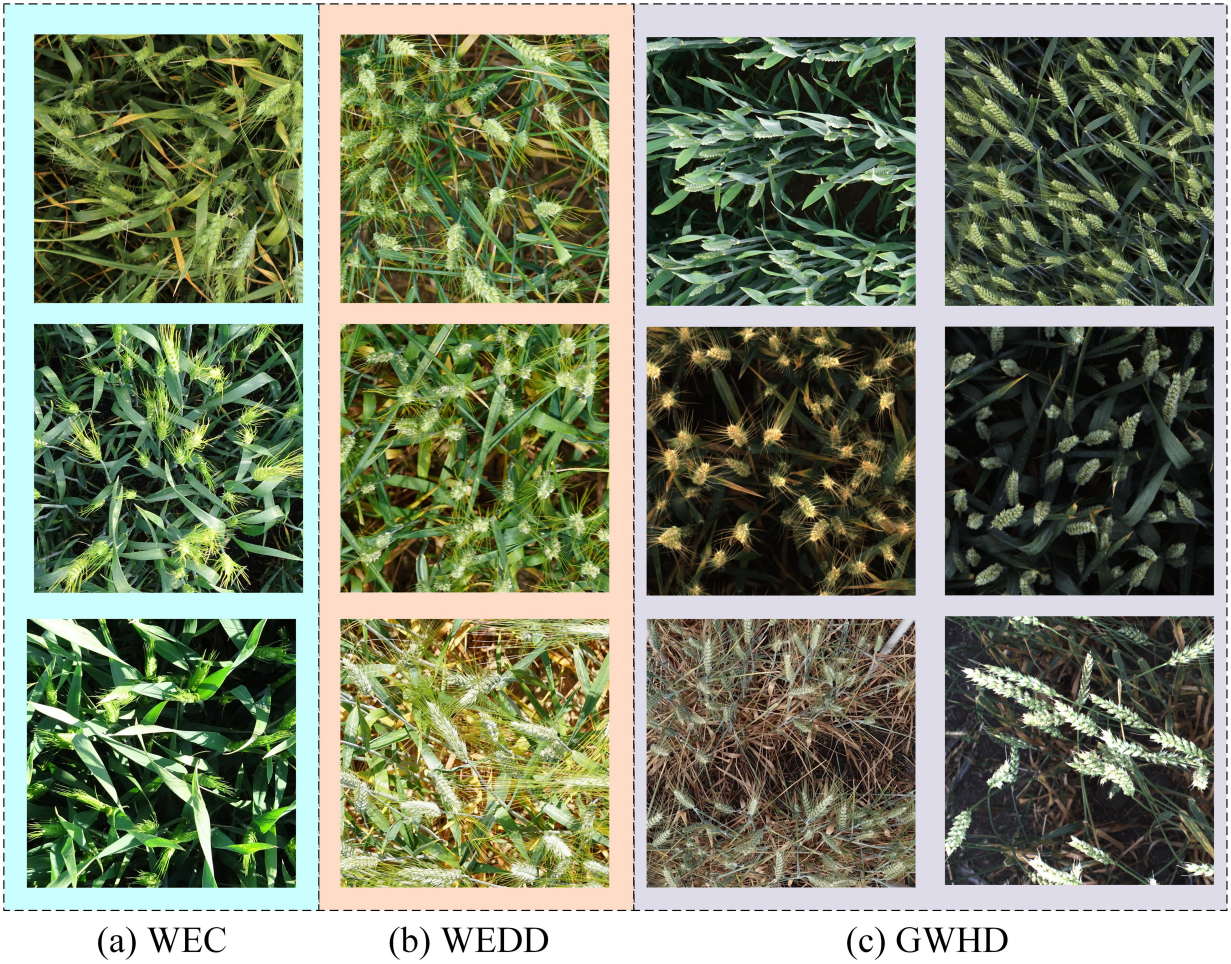
Examples of wheat image: **(A)** WEC, **(B)** WEDD, and **(C)** GWHD.

**Figure 1** shows some examples of wheat data sets, including three data sets, WEC, WEDD and GWHD. Among them, **Figure 1A** represents WEC data set, **Figure 1B** represents WEDD data set, and **Figure 1C** represents GWHD data set. There are certain differences in the shape, density, occlusion, and period of the wheat ears, which can intuitively reflect the diversity of wheat ears in the wheat data set constructed in this study.

#### 2.1.2 Data annotation

**Figure 2:**
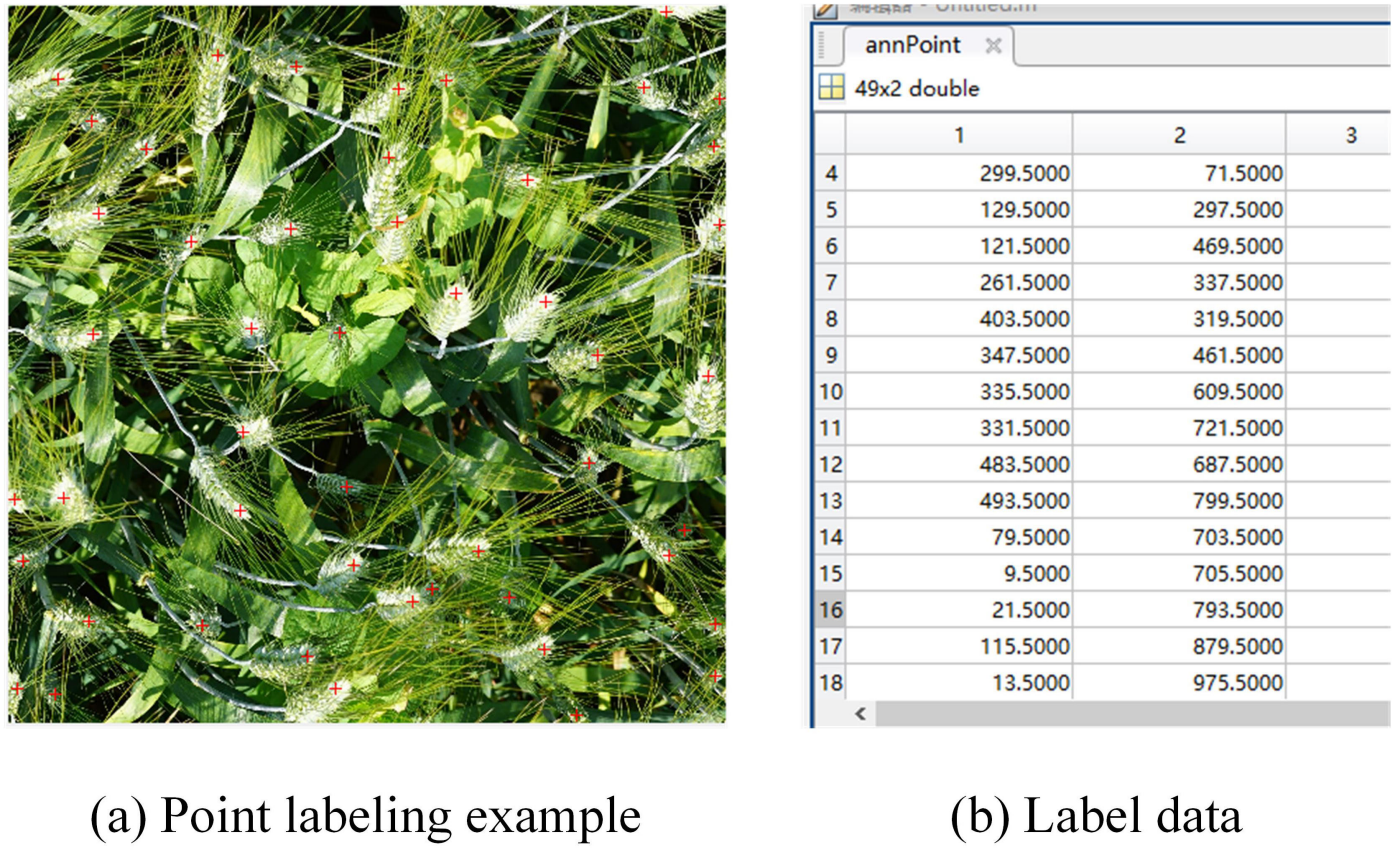
Example of wheat ear labeling: **(A)** Point labeling example, **(B)** Label data.

After the wheat data is selected, MATLAB is used to label the wheat ear points on the images in the above data set, as shown in **Figure 2A**. And **Figure 2B** shows the.mat format tag that saves the wheat ear position information. This annotation technology not only maintains the clarity of the image, but also more accurately captures the spatial position information of the wheat ears in the image. To further reduce the computational burden and annotation workload, all wheat images in the data set are uniformly adjusted to a high resolution of 1024 × 1024 pixels before annotation, which not only ensures the accuracy of annotation, but also improves processing efficiency.

#### 2.1.3 Dataset construction

The reasonable division of the wheat data set is a critical step for the subsequent model training and evaluation process. The density estimation model will be trained and tested based on the wheat data set divided in **Table 1**. The ratio of the training set, verification set, and test set is set to 7:2:1, and the detailed numbers are 840, 240, and 120 images respectively.

**Table 1:** Dataset details for this study.

Data set	Number of images	Training set	Validation set	Test set	Total number of wheat ears
WEC	500	350	100	50	12721
GWHD	500	350	100	50	22380
WEDD	200	140	40	20	11308
	1200	840	240	120	46409

### 2.2 GhostNet

**Figure 3:**
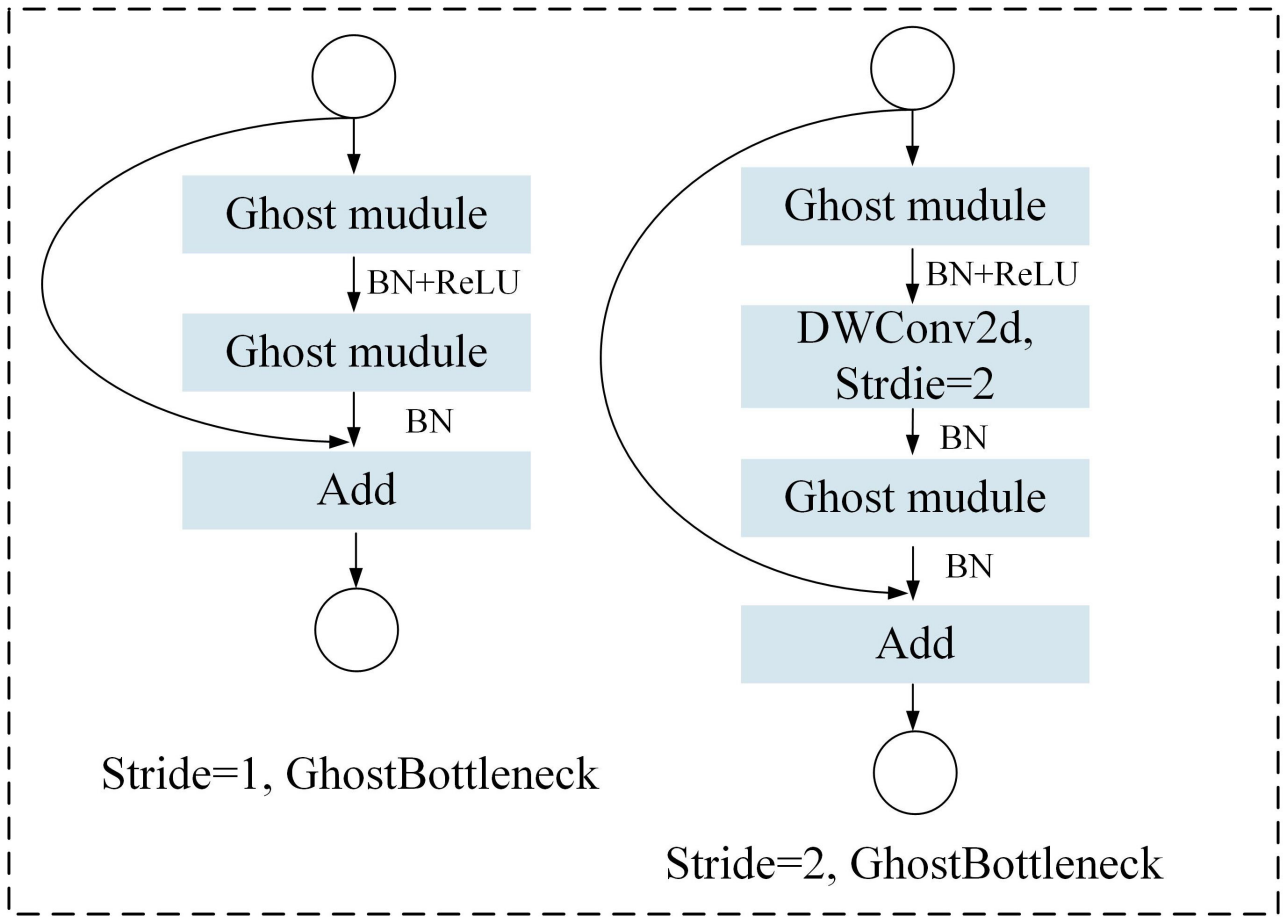
GhostNet model.

The GhostNet is a lightweight network framework based on the Ghost module, which is mainly composed of simple linear operations and standard convolutions (Han et al., 2019). Specifically, the Ghost module generates some intrinsic feature maps through ordinary convolutions, and then generates more feature maps from these feature maps through a series of simple linear operations. These new feature maps are combined with the original feature maps to form the final feature map output, as shown in **Figure 3**. This structure enables GhostNet to greatly reduce the size and number of parameters of the model while ensuring model performance, making the model more lightweight. The GhostBottleneck module is an important part of the GhostNet model, which is mainly composed of two Ghost modules. Each Ghost module is responsible for generating feature maps and adding features and channels through linear operations. This structure enables the GhostBottleneck module to further reduce the computational complexity of the model while maintaining feature extraction capabilities.

### 2.3 Upsampling method

#### 2.3.1 Interpolation algorithm

Bilinear Interpolation Upsampling is a widely used technique in image processing to enlarge the size of an image to a higher resolution (Soh et al., 2024). The core idea is to perform linear interpolation in two directions (usually horizontal and vertical) to estimate and fill the values ​​of the newly added pixels in the enlarged image. Compared with other more complex upsampling methods, the calculation of bilinear interpolation is relatively simple and easy to implement.

#### 2.3.2 Transposed convolution

Transposed Convolution (Faisal et al., 2023), as a commonly used upsampling method in deep learning, increases the size of feature maps by simulating the inverse process of convolution layer. Transposed convolution can effectively help the model recover high-resolution images or feature maps from low-resolution features. However, the computational complexity of transposed convolution is high, and the demand for computing resources and storage space is also large, which needs special consideration when designing large or complex deep learning models.

#### 2.3.3 Dense upsampling convolution

Dense Upsampling Convolution (DUC) is an upsampling technique that compensates for the loss in length and width by dividing the entire feature map and operating on the channel dimension. It is mainly used to increase the resolution of an image or feature map to restore it to its original size or a larger size, while trying to preserve and restore the details in the image (Zhou et al., 2022).

### 2.4 Density estimation model

#### 2.4.1 MCNN

**Figure 4:**
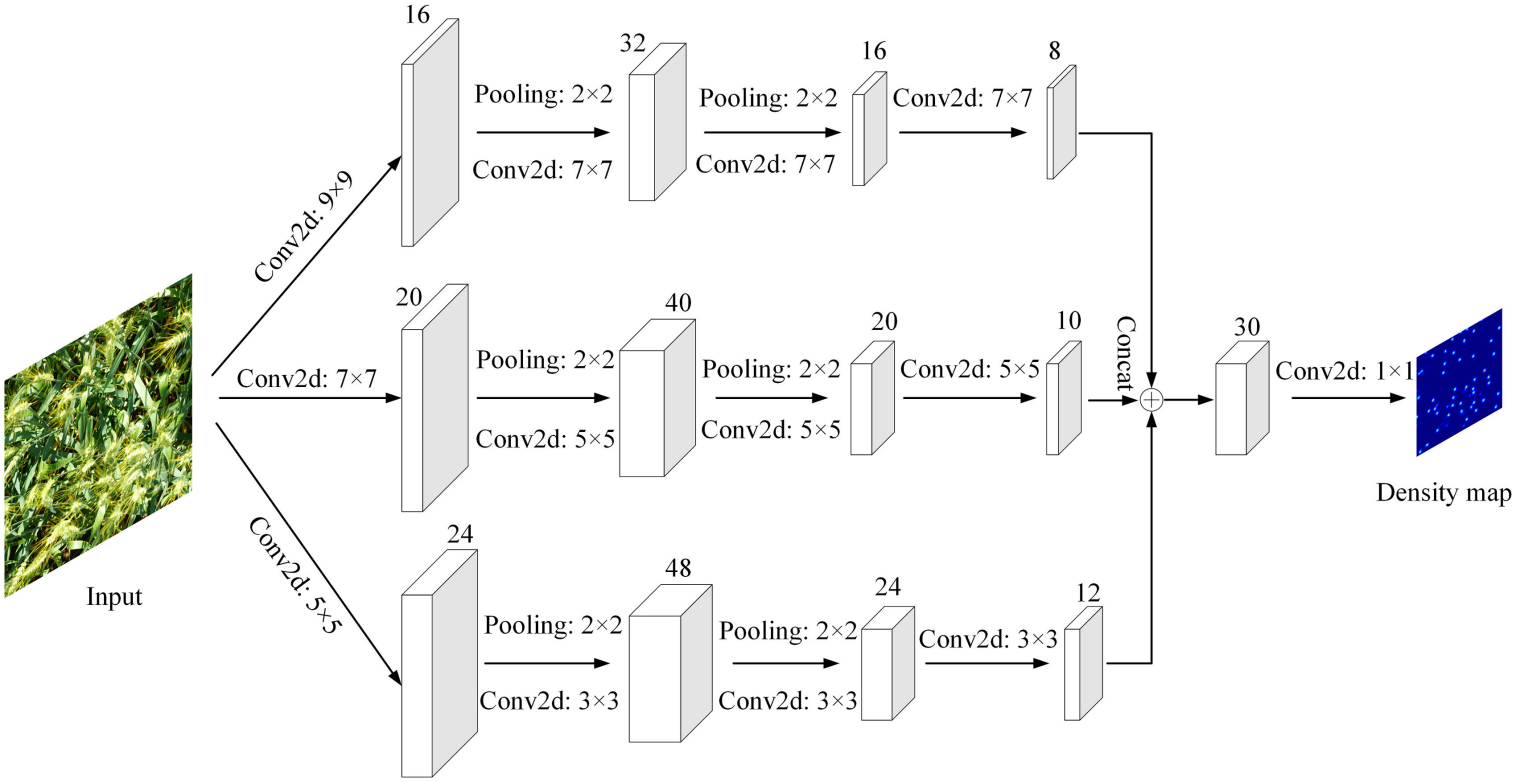
Network structure of MCNN model.

MCNN (Multi-column Convolutional Neural Network) is a three-column convolutional neural network model composed of three different convolution kernel sizes (Zhang et al., 2016). It completes the density estimation and counting tasks of the target by mapping the input image into a predicted density map. Wang et al. (2019) first applied it to wheat ear counting, using the feature map generated by the multi-column convolutional neural network for feature fusion, reducing the dimensionality of the fused features, and then outputting the predicted wheat ear density map and counting the wheat ears. The detailed network structure of MCNN is shown in **Figure 4**.

#### 2.4.2 CSRNet

**Figure 5:**
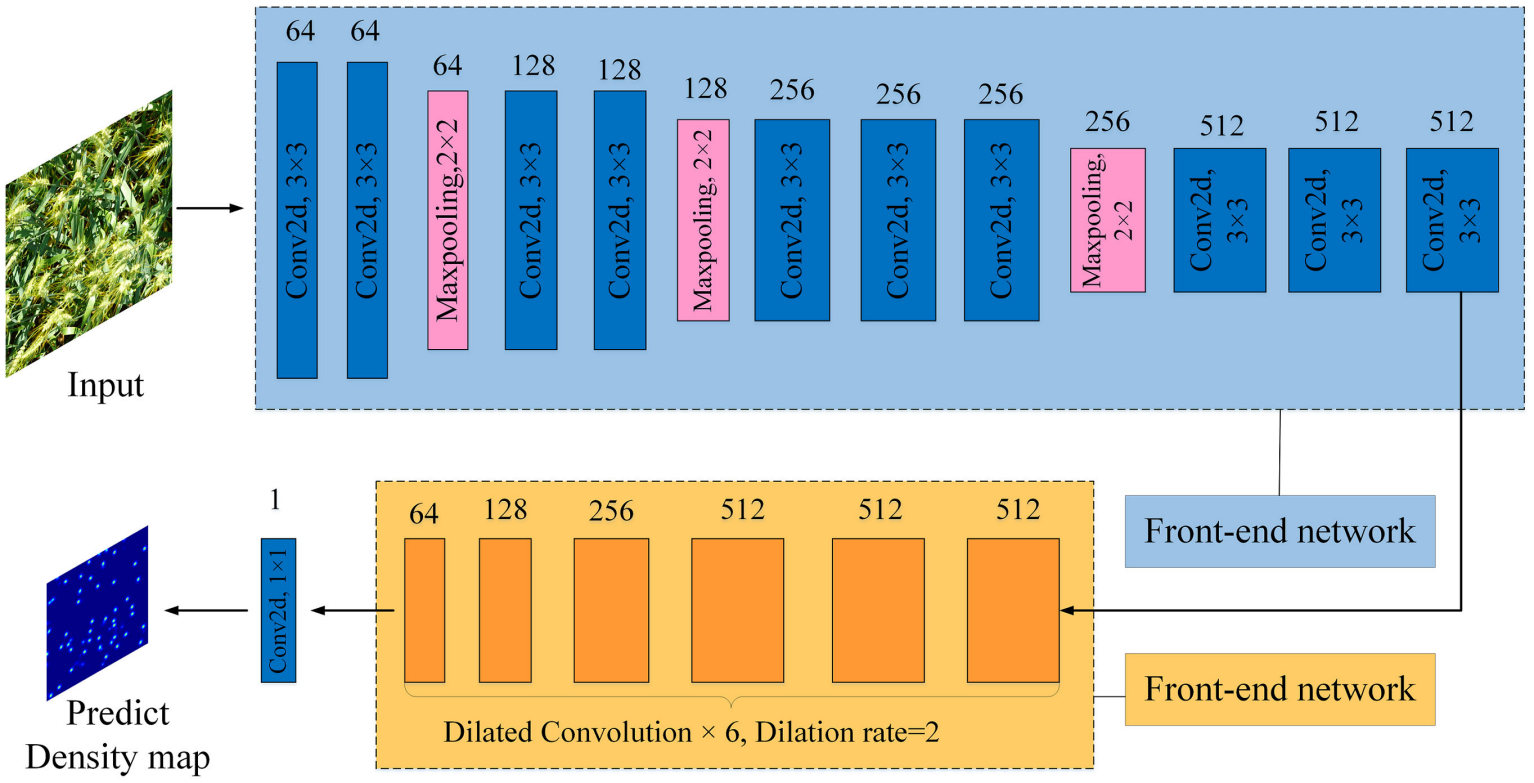
Network structure of CSRNet model.

CSRNet (Congested Scene Recogrition Network) is a target counting model suitable for dense scenes (Li et al., 2018). Its network structure mainly consists of a front-end network and a back-end network, which are VGG-16 with all fully connected layers removed, and 6 consecutive dilated convolution operations with the dilation rate set to 2. Finally, after a 1×1 ordinary convolution operation, the predicted density map is output and the targets are counted. The CSRNet model structure is shown in **Figure 5**.

#### 2.4.3 FIDTM

**Figure 6:**
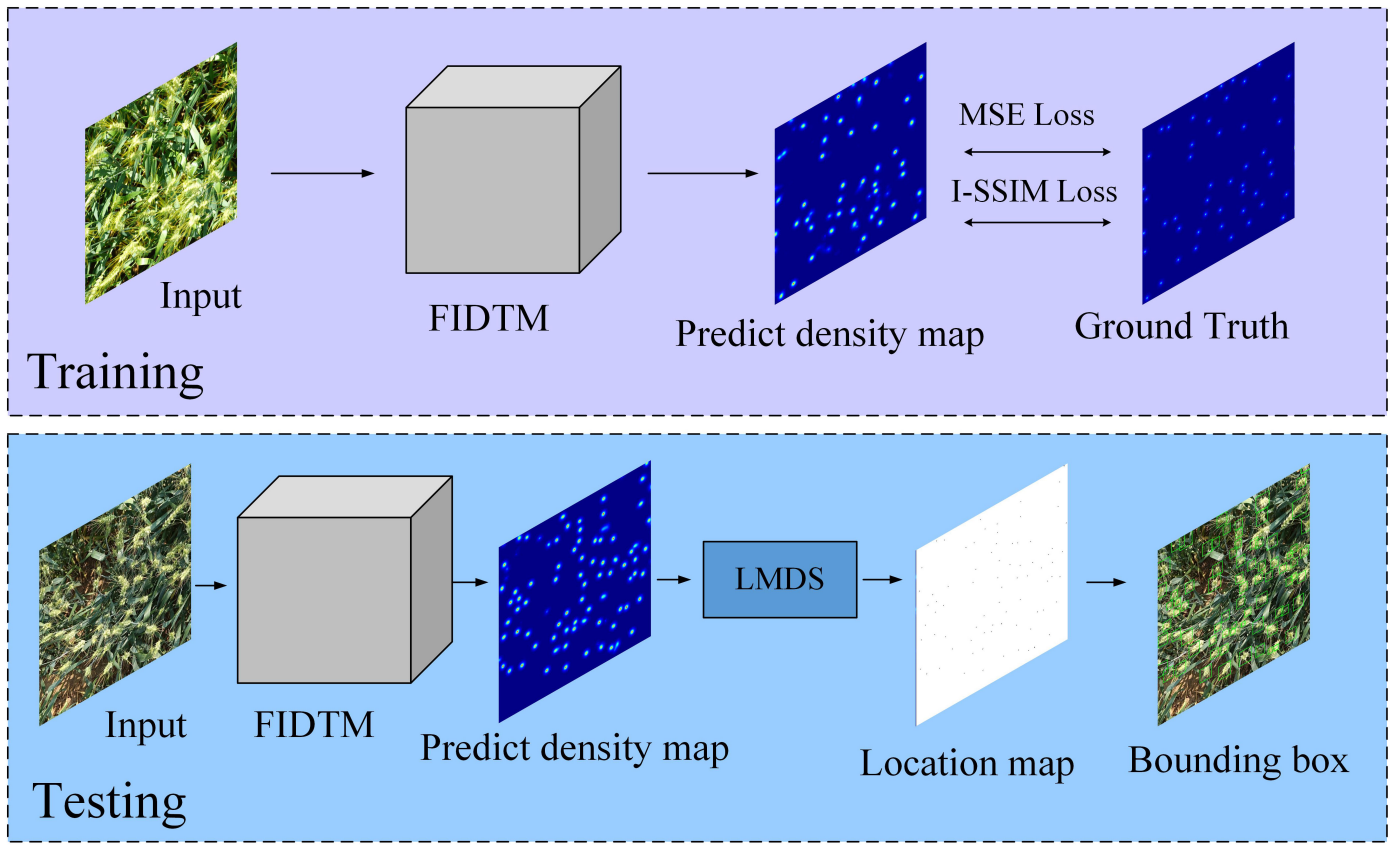
Wheat ear counting process based on FIDTM model.

FIDTM is a convolutional neural network model for density estimation and counting of dense targets (Liang et al., 2022). It is designed to solve the problem of accurately counting and locating targets in extremely dense scenes. When dealing with wheat ear counting tasks, the FIDTM model shows its unique advantages, as shown in **Figure 6**. To locate and count wheat ears more accurately, the original FIDTM model will be improved. On the one hand, the Ghost module is introduced. We replace the original convolutional layer of the FIDTM model with the Ghost module to reduce the computational complexity and parameter amount of the model while maintaining or improving the performance of the model. On the other hand, in view of the fact that the FIDTM model may lose the detailed information of wheat ears during the upsampling process, the Dense Upsampling Convolution (DUC) module is used to obtain more wheat ear features. Dense upsampling mainly divides the entire feature map into multiple parts with the same size as the input feature map to make up for the loss in length and width by increasing the channel dimension.

### 2.5 Positioning based on LMDS

The local maximum detection strategy (LMDS) is an important component in the wheat ear counting task, which is used to effectively extract the center point of each wheat ear. In wheat ear counting, the local maximum detection strategy works by identifying possible wheat ear regions through image segmentation or object detection algorithms. Then, within these wheat ear regions, the maximum values of local pixel intensity or feature values are found, which usually correspond to the center points of the wheat ears. Finally, these center points are extracted and the number of wheat ears in the image was accurately counted.

In this study, the point coordinates and K nearest neighbor distance of each wheat ear can be obtained by using the LMDS algorithm, and the approximate size of the wheat ear detection frame is defined as shown in Equation 1.


(1) $$D_{(x,y)\in P}=\min\{\begin{array}{c} \bar{d}=f\times \frac{1}{k}\sum \begin{array}{c} k\\ 1 \end{array}d_{\left(x,y\right)}^{k}\\ \min(img\_w,img\_h)\times 0.05 \end{array}$$

Where $D_{\left(x,y\right)}$ represents the size of the wheat ear detection box located at (x,y) , *P* represents the set of predicted wheat ear positions, $\bar{d}$ represents the average distance between the wheat ear point set $P_{\left(x,y\right)}$ and uses a scalar factor *f* to limit the size. In images with sparse wheat ears, $\bar{d}$ is generally much larger than the actual wheat ear size, so a threshold needs to be selected related to the image size to limit the wheat ear size.

### 2.6 Density map based on FIDT mapping

To accurately locate wheat ears in images in relatively dense areas, the new label of Focal Inverse Distance Transform (FIDT) of the target positioning task is used, that is, the position of each wheat ear is represented by the nearest neighbor distance information. The generation principle of the FIDT graph is based on the conversion graph of Euclidean distance (Sun et al., 2019). The conversion graph of Euclidean distance is defined as shown in Equation 2:


(2) $$P(x,y)=\min\sqrt{{\left(x-x^{'}\right)}^{2}+{\left(y-y^{'}\right)}^{2}}$$

In the formula, $(x^{'},y^{'})\subset \text{B}$ and B represents the set containing all wheat ear annotation information. For any pixel (x,y) in the image, P(x,y) represents the shortest distance between each pixel in the image and its nearest wheat ear center point.

At the same time, to make it easier to distinguish the foreground target from the background, the focus inverse distance transformation map is used in the FIDTM model, which is defined as:


(3) $$I=\frac{1}{P{\left(x,y\right)}^{\left(\alpha \times P(x,y)+\beta \right)}+C}$$

where *I* represents the FIDT diagram, *α* and *β* are set to 0.02 and 0.75 respectively in the FIDTM model.

### 2.7 Count of wheat ear density map based on FIDMT-GhostNet

#### 2.7.1 Overall technical route

**Figure 7:**
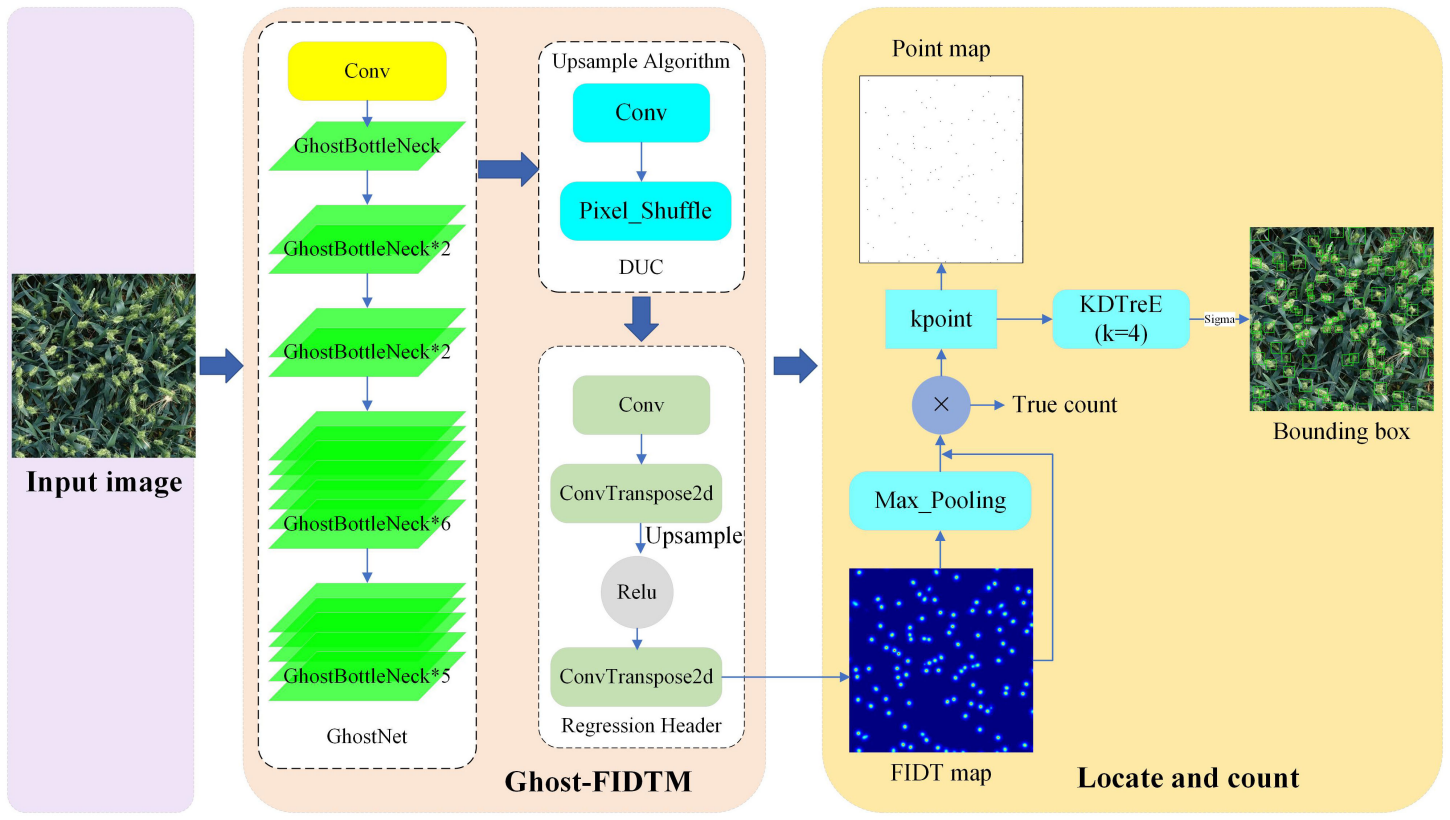
The flow chart of wheat ear counting based on the FIDMT-GhostNet.

The flow chart of wheat ear counting based on the FIDMT-GhostNet model is shown in **Figure 7**. Firstly, the wheat image is used as the input of the model, and then the feature map is obtained after 1 standard convolution and 16 consecutive GhostBottleneck modules, which is upsampled through the DUC algorithm to make the resolution of the feature map consistent with the input wheat ear image. Secondly, the wheat ear density map was predicted based on a standard convolution with a convolution kernel size of 1 × 1 and an output channel. Finally, the counting and positioning of wheat ears are realized based on the density map and LMDS algorithm. The wheat ears were located and detection frames were generated by predicting the local maximum of the density map. Among them, the target positioning information output by the FIDMT-GhostNet model is based on the pixel level, that is, only the position coordinates of the center point of the wheat ear are determined. The final result of the detection requires obtaining the wheat ear area in the image, that is, locating the target frame of each wheat ear. Therefore, the target frame of each wheat ear can be calculated using LMDS.

The FIDMT-GhostNet model innovatively introduces a continuous stacking structure of 16 GhostBottleneck modules when building its backbone feature extraction network. This strategy greatly enhances the model’s ability to perceive details and texture information of wheat ear images in a multi-level feature space, thereby significantly improving the accuracy of wheat ear counting. It is worth mentioning that despite the large number of modules, the efficiency of the GhostBottleneck module ensures that the model consumes reasonable computing resources, allowing FIDMT-GhostNet to maintain high performance while also achieving high computing efficiency. This design not only reflects the ingenuity of the model design, but also demonstrates the wisdom of seeking a balance between calculation accuracy and efficiency in the wheat ear counting task.

#### 2.7.2 Design of loss function

The loss function is used to calculate the error between the model output and the true result. The Euclidean distance loss $L_{e}$ and the density consistency loss $L_{c}$ are used as the loss *L* of the overall network, that is, the total loss *L* used by the network is the weighted superposition of $L_{e}$ and $L_{c}$ . The calculation formula is shown in Equation 4 and 5.


(4) $$L=L_{e}+L_{c}$$

(5) $$L_{e}=\sqrt{\frac{1}{N}{\sum_{i=1}^{n}\left|x_{i}^{GT}-x_{i}^{P}\right|}^{2}}$$

In the formula, *N* is the number of wheat ear samples, $x_{i}^{GT}$ and $x_{i}^{P}$ are respectively ground truth and the estimated wheat ear count corresponding to the *i*th wheat ear sample.

SSIM (Structural Similarity Index Measure) is a metric used to measure the structural similarity between two images, including the brightness, contrast, and structural information of the image. SSIM values range from -1 to 1, where 1 means the two images are identical, 0 means there is no similarity, and -1 means the two images are completely different. To comprehensively evaluate the performance of the FIDMT-GhostNet model in target counting and localization tasks, SSIM is used to evaluate the similarity between the predicted wheat ear density map and the ground-truth density map to evaluate the performance of the model.


(6) $$SSIM(E,G)=\frac{\left(2\mu_{E}\mu_{G}+\chi_{1})(2\sigma_{EG}+\chi_{2}\right)}{\left(\mu_{E}^{2}+\mu_{G}^{2}+\chi_{1})(\sigma_{E}^{2}+\sigma_{G}^{2}+\chi_{2}\right)}$$

(7) $$L_{c}=\frac{1}{N}\sum_{n=1}^{N}\left(1-SSIM(DM(I_{n}),\omega \right)$$

Among them, *N* is the total number of training samples, $I_{n}$ is the *n*th image input to the model, $DM(I_{n})$ is the predicted FIDT image obtained by inputting the *n*th image into the model, and *ω* is the FIDT image obtained by the focal inverse distance transform of the *n*th image. $\mu_{E}$ and $\sigma_{E}^{2}$ are the mean and variance of the predicted density map respectively, $\mu_{G}$ and $\sigma_{G}^{2}$ represent the mean and variance of the Ground Truth respectively. $\sigma_{EG}$ represents the covariance between the predicted density map and Ground Truth, $\chi_{1}$ =0.0001, $\chi_{2}$ =0.0009. The value range of SSIM is between -1 and 1. The larger the value, the more similar the two images are.

#### 2.7.3 Density estimation evaluation index

In density estimation, root mean squared error (RMSE) is often used to measure the difference in each pixel value between the predicted density map and the true density map. The mean absolute error (MAE) calculates the average of the absolute value of the difference between the predicted value and the true value. Compared with MSE, MAE is more robust to outliers. To evaluate the wheat ear counting performance of FIDMT-GhostNet, MAE, RMSE, and $R^{2}$ are used as evaluation indicators of the model (Qiao et al., 2023; Zhang et al., 2022b).


(8) $$MAE=\frac{1}{N}\sum_{i=1}^{n}\left|x_{i}^{GT}-x_{i}^{P}\right|$$

(9) $$RMSE=\sqrt{\frac{1}{N}{\sum_{i=1}^{n}\left|x_{i}^{GT}-x_{i}^{P}\right|}^{2}}$$

(10) $$R^{2}=1-\frac{\sum_{i=1}^{N}\left(x_{i}^{GT}-x_{i}^{P}\right)}{\sum_{i=1}^{N}\left(x_{i}^{GT}-{\bar{x}}_{i}^{P}\right)}$$

In the formula, *N* is the number of tested wheat ear samples, ${\text{x}}_{i}^{GT}$ and $x_{i}^{P}$ are respectively the Ground Truth and estimated wheat ear count corresponding to the *i*th wheat ear sample, *MAE* can directly reflect the accuracy of the wheat ear counting model, and *RMSE* can better reflect Out of the robustness of the wheat ear counting model, $R^{2}$ reflects the degree of fit between the predicted value of the wheat ear and the Ground Truth. The closer the value is to 1, the higher the degree of fitting and the higher the reliability of the trend line.

In addition, parameters (Parameter), Floating Point Operations (FLOPs), Model Size, and FPS (Frames Per Second) indicators are also introduced to evaluate the wheat ear counting efficiency of the model.

## 3 Results

### 3.1 Experimental parameter settings

In this study, the PyTorch deep learning framework is used to build the network model. The system information and other software and hardware related information are listed in **Table 2**.

**Table 2:** Details of experimental hardware and software parameters.

Configuration name	Parameters
Operating System	Windows 10 Professional 64-bit
Code running environment	Python3.7
Deep learning framework	Pytorch1.80
GPU model	NVIDIA GeForce RTX 2080
Processor	Intel Core i7-8700 CPU@3.20GHz

The parameter settings during model training are shown in **Table 3**. When the Epoch value is greater than or equal to 100, verification is performed every 5 Epoch intervals.

**Table 3:** Training parameters of FIDMT-GhostNet model.

Parameter name	Value
Epoch	1000
Batch size	2
Learning rate	0.0001
Weight decay	0.0005
Optimizer	Adam

### 3.2 Wheat ear counting results

To verify the effectiveness and feasibility of counting method proposed for the wheat ear in this study, and to evaluate the universality and transferability of the FIDMT-GhostNet model, we used WEC, GWHD, and WEDD data sets to carry out wheat ear counting. The counting results are shown in **Table 4**. From the detailed data in **Table 4**, we can observe the counting performance of the model under different experimental data. An in-depth analysis of these performance metrics reveals several noteworthy trends or characteristics. Among the three data sets, the FIDMT-GhostNet model performs best with the WEC data set. In particular, the WEC data set test has the lowest MAE and RMSE, and the highest R^2^, reaching 3.25, 3.56, and 0.9279 respectively. Compared with the GWHD and WEDD data sets, the MAE of WEC decreased by 2.23 and 1.62 respectively, the RMSE decreased by 2.67 and 2.02 respectively, and the R^2^ increased by 0.1153 and 0.0067 respectively. The generalization performance of the FIDMT-GhostNet model on the GWHD and WEDD test sets is lower than that on the WEC test set. It may be that the GWHD data set comes from seven different countries, and there are certain differences in the size, shape, and color of wheat ears. The wheat ears in the WEDD data set are more densely distributed and have a higher degree of occlusion. However, the wheat ears in the WEC data set have a low degree of occlusion, and the differences between wheat ears are small. Therefore, the optimized FIDMT-GhostNet model has demonstrated excellent counting capabilities in multiple data sets. Especially when processing wheat ear images with severe occlusion and complex backgrounds, the model can still maintain a high counting accuracy. This further verifies the effectiveness and robustness of the model design.

**Table 4:** Wheat ear count results from different datasets.

Dataset	Number of images	MAE	RMSE	R^2^
WEC	50	3.25	3.56	0.9279
GWHD	50	5.48	6.23	0.8126
WEDD	20	4.87	5.58	0.9212

**Figure 8:**
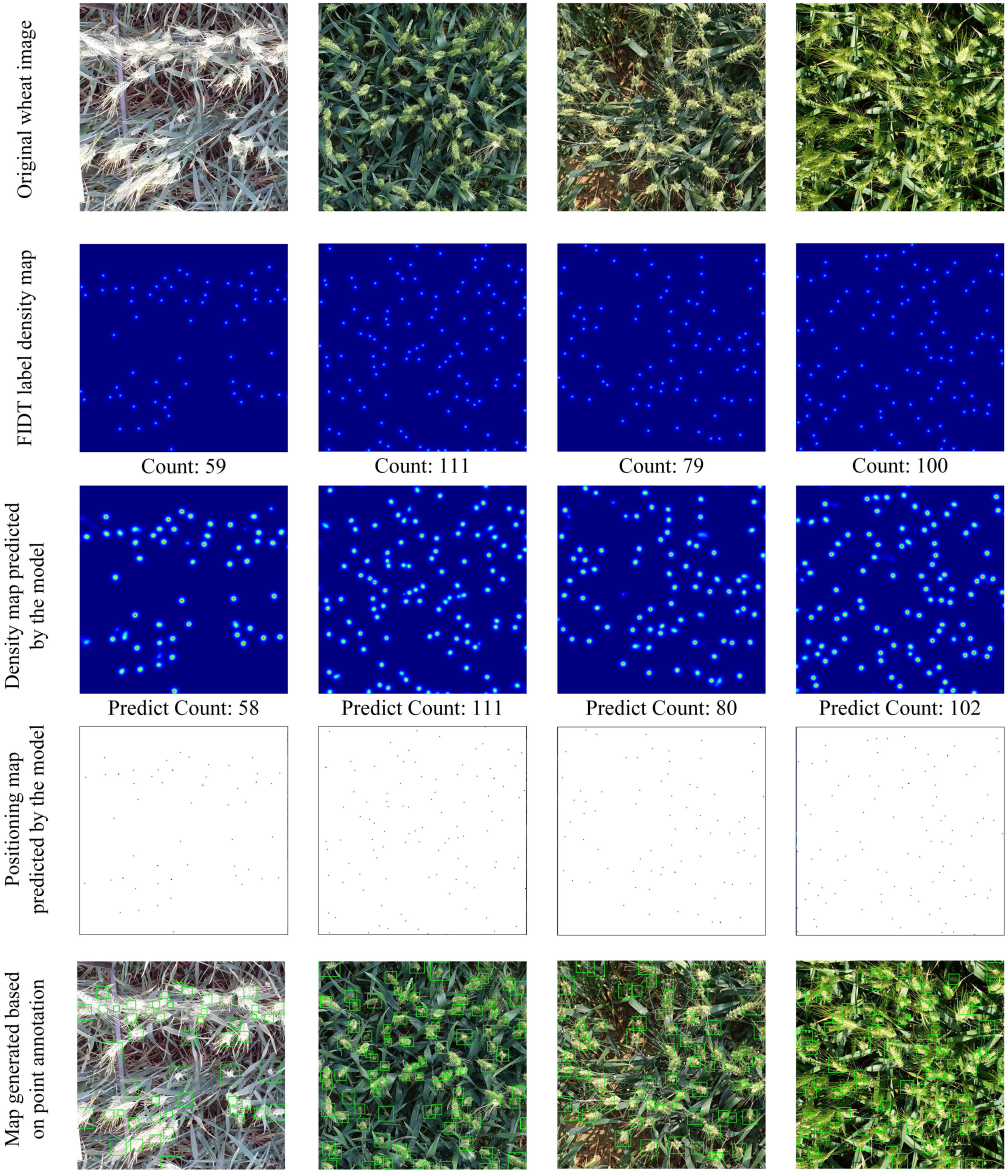
Results of wheat ear counts from FIDMT-GhostNet model.

To further prove the wheat counting performance of the FIDMT-GhostNet model, **Figure 8** shows the density plot of the output of some samples of the data set. These sample data come from different data sets and wheat ears from different countries. It can be seen from the wheat ear counting results in **Figure 8** that this model has certain universality and generalization in the wheat ear counting task.

In the wheat image analysis shown in **Figure 8**, we can observe that from top to bottom are the original wheat image, the FIDT label density map, the density map predicted by the model, the wheat ear positioning map predicted by the model, and the map generated based on point annotation. Although there are significant differences in the distribution, occlusion degree, shape and growth cycle of the wheat ears in the test image, the density map predicted based on the FIDMT-GhostNet model and the FIDT label density map show a high degree of consistency in distribution, which fully demonstrates that the FIDMT-GhostNet model has the ability to handle differences between wheat ears. Furthermore, for the wheat ears in the original image, the wheat ear counting results of the FIDMT-GhostNet model is very close to the ground truth, which shows not only the accuracy of the model in the counting task, but also its reliability and effectiveness in practical applications. In particular, the wheat ear detection frame predicted based on the FIDMT-GhostNet model can more accurately cover the complete wheat ear, which further verifies the performance of the model in positioning and scale estimation. Therefore, the test results of different data sets show that the proposed model can not only achieve the task of counting wheat ears, but also obtain accurate position and scale information of wheat ears.

## 4 Discussion and analysis

### 4.1 Counting results of wheat ears with different degrees of occlusion

**Figure 9:**
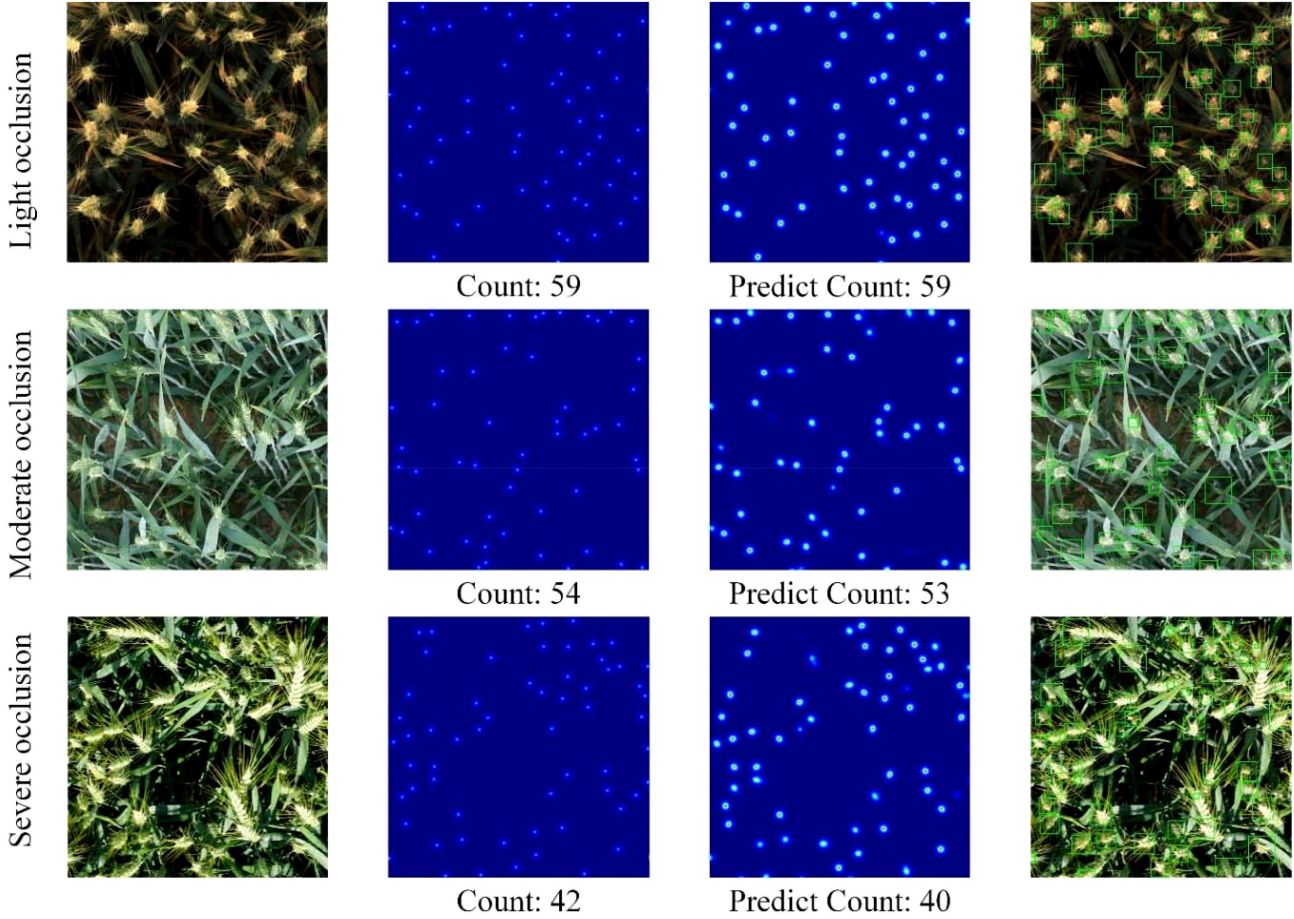
Counting results of wheat ears with different occlusion levels.

Wheat ears in the field often show varying degrees of occlusion due to overlapping in the middle and late stages of growth, which increases the difficulty of automatic counting. Occlusion will not only cause some wheat ears to be difficult to identify in the image, but may also affect the accuracy and stability of the counting algorithm. To evaluate the wheat ear counting effect of the proposed FIDMT-GhostNet model under different degrees of occlusion, wheat images with mild occlusion, moderate occlusion and severe occlusion were specially selected for testing in the test set, aiming to intuitively demonstrate the model’s ability to count different Adaptability to occlusion situations and counting performance. **Figure 9** shows the test results of the model. From left to right, they are the wheat ear image, density map of FIDT label generated using point annotation, the predicted density map, and positioning map of wheat ear.

As can be seen from **Figure 9**, the prediction results of wheat ear counting are very close to ground truth, and the prediction of wheat ear detection frame can accurately locate most wheat ears. The FIDMT-GhostNet model shows excellent counting capabilities in the wheat ear counting task, mainly due to the SE-block module embedded in its structure. By introducing an adaptive feature recalibration mechanism, SE-block significantly enhances the model’s ability to express features of important channels while suppressing the influence of useless channels. In wheat ear counting tasks, occlusion between wheat ears is a common and thorny problem. However, SE-block can enhance the model’s focus on important channels, allowing the model to more accurately identify occluded wheat ear features. At the same time, by suppressing the influence of useless channels, the FIDMT-GhostNet model can reduce interference caused by background noise or other irrelevant features, further improving the accuracy of wheat ear counting. As shown from the first row in **Figure 9**, the wheat ears have higher definition in the image, and there is almost no occlusion. The output of the density map is basically the same as the ground truth density map. As shown in the second and third rows of **Figure 9**, the FIDMT-GhostNet model can handle the scene where wheat leaves block wheat ears to a certain extent. Even in the case of overlapping wheat ears, the wheat ear density map estimated using the FIDMT-GhostNet model has a smaller error than the ground truth density map. The test results show that the wheat attention mechanism in the model plays an indispensable role. It enables the model to automatically identify and focus on the wheat ear area in the image, effectively suppressing the interference of background noise and other irrelevant elements. This mechanism not only improves the robustness of the model, but also enables the model to maintain stable performance in complex and changeable field environments.

### 4.2 Wheat ear counting results with different upsampling algorithms

To verify the effectiveness of the dense upsampling algorithm used in this article, three different upsampling algorithms were designed, including GhostNet+bilinear interpolation, GhostNet+dense upsampling, and GhostNet+deconvolution. When the same parameters are set during model training and testing, the test results are shown in **Table 5**.

**Table 5:** Counting results of wheat ears by different upsampling algorithms.

Upsampling algorithm	MAE	RMSE	R^2^
GhostNet + bilinear interpolation	4.72	6.16	0.8423
GhostNet + deconvolution	4.58	6.02	0.8815
GhostNet + dense upsampling	4.46	5.87	0.9145

It can be seen from **Table 5** that when the backbone feature network is GhostNet, the wheat ear counting effect is the best when the model uses dense upsampling convolution, and its MAE, RMSE, and R^2^ are 4.46, 5.87, and 0.9145. Compared with using the bilinear interpolation algorithm and deconvolution operation, the MAE of the model with dense upsampling convolution was reduced by 0.26 and 0.12 respectively, the RMSE was reduced by 0.29 and 0.15, and the R^2^ was increased by 0.0722 and 0.033, respectively.

In fact, the bilinear interpolation algorithm may cause the loss of some wheat ear features. Because bilinear interpolation estimates the value of the middle pixel based on the values of the surrounding four pixels, this method performs better when dealing with smoothly changing areas. However, when dealing with complex structures and edge information like wheat ears, the bilinear interpolation algorithm may lose some subtle features, thereby affecting the accuracy of wheat ear counting. The deconvolution operation may indeed produce some unnecessary overlap and aliasing, resulting in image blur and distortion. This is because the deconvolution process is essentially a convolution operation with special parameter settings. If the parameter settings are improper or the convolution kernel design is unreasonable, the above problems may occur. In addition, deconvolution itself will introduce a lot of matrix multiplications during backpropagation, which may indeed increase the risk of gradient disappearance or explosion, thereby affecting the training and testing effects of the model. The dense upsampling algorithms allows the model to recover more spatial detail information, which plays a crucial role in accurately locating and counting wheat ears. This algorithm effectively improves the image resolution, enhances the model’s ability to identify wheat ears, and further optimizes the counting results.

### 4.3 Comparison of wheat ear counting results based on different models

To evaluate the wheat ear counting performance of the FIDMT-GhostNet model proposed in this article, we selected three other density estimation models based on convolutional neural networks to compare with the model proposed in this article, including MCNN (Zhang et al., 2016), CSRNet (Li et al., 2018), and FIDTM (Liang et al., 2021). The data used for training and testing of these three models are distributed according to **Table 1**, while ensuring that the remaining parameter settings remain consistent to obtain the test results of wheat ear counting. The results of wheat ear counting are shown in **Table 6**. Among them, the leftmost column shows several classic counting network models and our model. The second, third and fourth columns are MAE, RMSE and R^2^.

**Table 6:** Wheat ear count results for different models.

Model	MAE	RMSE	R^2^
MCNN	8.51	12.48	0.4844
CSRNet	7.27	8.69	0.7099
FIDTM	4.39	6.24	0.8632
Ours	4.46	5.87	0.9145

As can be seen from **Table 6**, the MAE of FIDMT-GhostNet reaches 4.46, which is 4.05, 2.81, lower than that of MCNN, CSRNet, respectively, and is close to that of FIDTM. The RMSE of FIDMT-GhostNet is 5.87, which is 6.61, 2.82, and 0.37 lower than that of MCNN, CSRNet, and FIDTM. The R^2^ of FIDMT-GhostNet is 0.9145, which is 0.4301, 0.2046, and 0.0513 higher than that of MCNN, CSRNet, and FIDTM. Therefore, experimental results show that our model outperforms other models on multiple evaluation metrics.

We believe that this result may be due to the following two reasons. On the one hand, the irregular distribution of wheat ears and the large differences in size, shape, density, aspect ratio, etc. of wheat ears make it difficult for MCNN and CSRNet networks to generate high-quality label density maps through Gaussian kernel functions. Of course, in future research, the settings of the multi-column convolution kernel of the MCNN model and the hole rate of CSRNet need to be further optimized to better extract the effective features of wheat ears, thereby improving the accurate counting of wheat ears.

On the other hand, we believe that the good performance of FIDMT-GhostNet comes from the introduction of the Ghost module and the dense upsampling convolution module, which enables the network to adapt to the diversity of wheat ears and focus on wheat ears more effectively. In addition, the FIDTM network can solve the current situation of wheat ear scale changes and unbalanced density distribution. We also found that the MAE and RMSE of FIDMT-GhostNet model test results were reduced by 63% and 48.04% respectively compared to CSRNet, and R^2^ increased by 22.37%. The possible reason is that the backend of the CSRNet model uses atrous convolution to expand the receptive field, which affects the extraction of small target features of wheat ears. It may be that the atrous convolution in the back end of the CSRNet model expands the receptive field, which affects the extraction of small target features of wheat ears.

**Figure 10:**
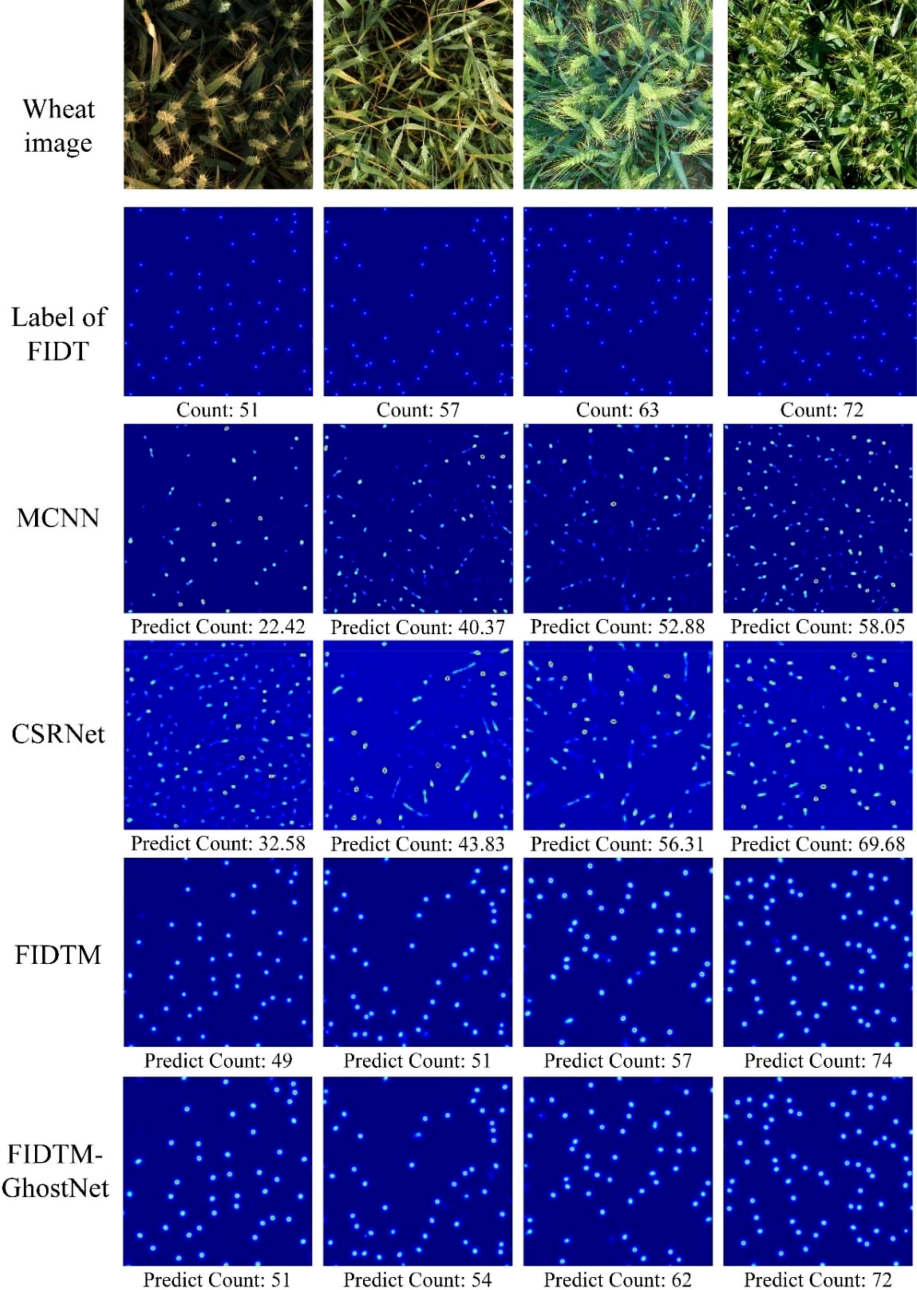
Results of wheat ear counts for different models.

**Figure 10** shows random samples of specific density estimation results for the above four models. The wheat ear counting results of the four density estimation models in **Figure 10**. We found that the wheat ear density map predicted based on these four models compared with the ground truth label density map, the errors from large to small are MCNN, CSRNet, FIDTM and FIDMT-GhostNet, respectively. Among them, the number of wheat ears predicted based on the FIDMT-GhostNet model is closest to the ground truth. In the third row of **Figure 10**, the wheat ear density map estimated based on MCNN was showed. In this part, we see that the estimated density map in the third row is quite different from the real density map in the second row, which indicates that the wheat ear counting effect of the multi-column convolutional neural network MCNN is poor. The possible reason is that MCNN has network structure redundancy, which results in low feature extraction efficiency. Although it has successfully overcome problems such as target occlusion and scale differences to a certain extent, it is difficult to effectively extract the global features of complex wheat field scenes. To solve the above problems, Li et al. (2018) introduced a single-column convolutional neural network CSRNet, which uses dilated convolution to expand the receptive field. In particular, as the depth of the convolutional neural network deepens, the performance of the network structure will gradually increase. Compared with the multi-column structure, the single column convolutional neural network has certain advantages.

As can be seen from **Figure 10**, the detection accuracy of CSRNet model with the single column is higher than that of MCNN model with multi-column structure. At the same time, we also provide the density map estimated using FIDMT-GhostNet in the sixth row of **Figure 10**, which shows that the FIDMT-GhostNet model using the dense upsampling algorithm has better results in counting wheat ears in the field. In summary, compared with other common density map models, the FIDMT-GhostNet model proposed in this article has the highest accuracy and the lowest RMSE, indicating that it has good robustness.

### 4.4 Performance comparison of different models

**Table 7** shows the performance parameters of the four models. Among them, the Parameter, FLOPs, Model Size, and FPS of the proposed FIDMT-GhostNet reached 8.42M, 134.09G, 96.8568MB, and 9.26 respectively. Compared with CSRNet, the Parameter of our model is reduced by 48.2% and the FLOP is reduced by 69.1%. Previous studies have shown that Bao et al. (2020) used CSRNet to count wheat. The poor performance of wheat ear counting based on the CSRNet model is mainly due to its deep network structure, which results in a large number of parameters. Although this design can capture more complex wheat ear features in images, it may also cause problems such as high computational complexity, difficulty in model training, and overfitting. Therefore, when counting wheat ears in the field, it is necessary to weigh the balance between the depth and performance of the network to achieve a more efficient wheat ear counting task.

**Table 7:** Wheat ear count results for different models.

Model	Parameter (M)	FLOPs (G)	Model Size (MB)	FPS
MCNN	0.13	28.23	0.5186	52.63
CSRNet	16.26	433.36	62.0512	8.92
FIDTM	66.58	569.59	763.5506	3.13
Ours	8.42	134.09	96.8568	9.16

M/G in the table represent 10^6^/10^9^ respectively.

According to the wheat ear counting efficiency evaluation indicators in **Table 7**, MCNN performs well for the Parameter, FLOPs, Model Size, and FPS performance evaluation results of the model. On the one hand, MCNN removes the fully connected layer, resulting in reduced network parameters and a simple structure. In the experimental results, MCNN has the smallest Parameter, reaching 0.13M. Model Size reaches 0.5186MB. On the other hand, the MCNN model contains multiple columns of networks, so network training takes longer than an end-to-end network. This results in the performance indicators of wheat ear counting results based on MCNN, including MAE, RMSE, and R^2^, being worse than the other three models.

In addition, it can be seen from **Table 7** that the number of parameters in the FIDTM model is 66.58M, and its FLOPs are 569.59G. The parameter size of lightweight FIDMT-GhostNet is 8.42M, and the FLOPs are 134.09G. Compared with the original model, the Parameter, FLOPs and FPS of our proposed lightweight model were reduced by 87.4%, 76.5% and 87.3% respectively. In fact, the FIDTM model has good robustness in scenarios with unbalanced density distribution, but it counts wheat ears evenly distributed in the field. After extensive compression, some parameters in the model were removed, which improved the generalization ability of the FIDMT-GhostNet model, increasing R^2^ by 5.6% and reducing RMSE by 5.9%.

## 5 Conclusions and future work

In this study, an automatic positioning and counting method based on FIDMT-GhostNet was proposed to address the challenges faced by counting wheat ears, including complex backgrounds, dense ears, and different sizes. This method achieves precise positioning and counting of wheat ears through multi-scale feature extraction and point labeling networks, combined with dense upsampling and local maximum detection strategies. Three wheat ear databases including WEC, WEDD and GWHD were used for model training and testing. Experimental results show that the FIDMT-GhostNet model achieved high accuracy on the wheat image data set, and the number of parameters was small. RMSE and R^2^ reached 5.87 and 0.9145 respectively, and the number of parameters and FPS reached 8.42M and 9.16 respectively. Therefore, the experimental results show that the FIDMT-GhostNet has good robustness for wheat ear counting. With the continuous advancement of technology, the wheat ear counting model based on FIDMT-GhostNet is expected to be directly deployed to edge devices and play a greater role in precision agriculture with fewer parameters, faster inference speed, and good counting performance.

To further enhance the robustness and generalization ability of the model, we plan to continue to collect more wheat datasets in the future. These new datasets will not only focus on the increase in quantity, but also emphasize the diversity of data, covering wheat images of different varieties, different growth stages, and different environmental conditions, to ensure that the model can perform well in various practical scenarios.

GhostNet has achieved remarkable results in reducing the computational requirements of the model with its efficient network structure. To ensure that this combined model can be widely used in real-world scenarios, especially in resource-constrained agricultural environments, we will conduct in-depth analysis and optimize its computational efficiency. At the same time, in view of the diversity of agricultural image data scale and complexity, exploring how to adjust the model structure to better adapt to these data and enhance the scalability of the model will become a key direction for future research. To this end, we can use model compression and acceleration techniques, such as parameter pruning, quantization, and hardware acceleration, to further optimize the performance of the combined model. These advanced technologies can significantly reduce the storage occupancy and computational requirements of the model without significantly sacrificing model performance, thereby providing strong support for the widespread application of the combined model in resource-constrained agricultural environments.

## Data Availability

The original contributions presented in the study are included in the article/supplementary material. Further inquiries can be directed to the corresponding author.
